# Autophagy and Stem Cells: Self-Eating for Self-Renewal

**DOI:** 10.3389/fcell.2020.00138

**Published:** 2020-03-04

**Authors:** Natasha C. Chang

**Affiliations:** Department of Biochemistry, Faculty of Medicine, McGill University, Montreal, QC, Canada

**Keywords:** autophagy, stem cells, self-renewal, quiescence, mitochondria, aging, reprogramming, cancer stem cell

## Abstract

Autophagy is a fundamental cell survival mechanism that allows cells to adapt to metabolic stress through the degradation and recycling of intracellular components to generate macromolecular precursors and produce energy. The autophagy pathway is critical for development, maintaining cellular and tissue homeostasis, as well as immunity and prevention of human disease. Defects in autophagy have been attributed to cancer, neurodegeneration, muscle and heart disease, infectious disease, as well as aging. While autophagy has classically been viewed as a passive quality control and general house-keeping mechanism, emerging evidence demonstrates that autophagy is an active process that regulates the metabolic status of the cell. Adult stem cells, which are long-lived cells that possess the unique ability to self-renew and differentiate into specialized cells throughout the body, have distinct metabolic requirements. Research in a variety of stem cell types have established that autophagy plays critical roles in stem cell quiescence, activation, differentiation, and self-renewal. Here, we will review the evidence demonstrating that autophagy is a key regulator of stem cell function and how defective stem cell autophagy contributes to degenerative disease, aging and the generation of cancer stem cells. Moreover, we will discuss the merits of targeting autophagy as a regenerative medicine strategy to promote stem cell function and improve stem cell-based therapies.

## Autophagy: a Fundamental Cell Survival Mechanism

Autophagy is a fundamental cellular process by which cells sequester intracellular constituents, including organelles and proteins, that are delivered to lysosomes for degradation and recycling of macromolecule precursors (Galluzzi et al., 2017). The process of autophagy is evolutionarily conserved from yeast to mammals and serves as an essential adaptation mechanism to provide cells with a source of energy during periods of nutrient deprivation and metabolic stress. Under homeostatic conditions, cells maintain a constitutive basal level of autophagy as a method of turning over cytoplasmic content. Autophagy can also be induced in response to cellular stresses such as nutrient deprivation, oxidative stress, DNA damage, endoplasmic reticulum stress, hypoxia, and infection.

The term autophagy, which literally translates to “self-eating” was invented by Christian de Duve in 1963 to describe the presence of double-membraned vesicles containing cytoplasmic constituents within the cell (Klionsky, 2008; Yang and Klionsky, 2010). These vesicles that encapsulate cytoplasm, organelles and proteins, are known as autophagosomes and are the phenotypic hallmark of macroautophagy, the most prevalent and extensively characterized form of autophagy. Macroautophagy, which is the focus of this review, will hereby be referred to as autophagy.

**FIGURE 1:**
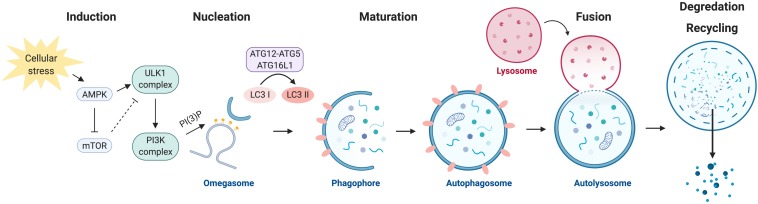
The autophagy program. Autophagy is negatively regulated by mTOR and positively regulated by AMPK. Activation of autophagy involves the formation of the ULK1 and PI3K protein complexes. Nucleation of the autophagosome structure involves the generation of PI(3)P at the omegasome. Maturation and elongation of the autophagosome membrane involves two ubiquitin-like conjugation steps, the conjugation of ATG12 to ATG5 and the conversion of LC3 I to LC3 II. Fully formed autophagosomes fuse with lysosomes to degrade their intracellular content and recycle macromolecule components.

Autophagy is a multi-step process of sequential events including induction, nucleation of a phagophore structure, maturation of the autophagosome, autophagosome fusion with the lysosome, and the degradation and recycling of nutrients (Figure 1) (Mizushima, 2007). The execution of autophagy is dependent on the formation of several key protein complexes and two ubiquitin-like conjugation steps. Initial studies performed to characterize key players in the autophagy pathway were carried out in yeast and identified a family of autophagy-related genes, referred to as *Atg*, which encode for autophagy effector proteins (Tsukada and Ohsumi, 1993).

Autophagy is negatively regulated by the nutrient-sensing mammalian target of rapamycin (mTOR) kinase, a master regulator of cellular growth and metabolism. Under nutrient-rich conditions, mTOR inhibits autophagy by preventing the formation of the autophagy initiation ULK1 Ser/Thr kinase protein complex. mTOR represses autophagy by direct phosphorylation of ULK1 (the mammalian ortholog of yeast Atg1) at serine residue (Ser) 757. Phosphorylation of ULK1 by mTOR at this site prevents ULK1 from interacting with a second master regulator of metabolism, the energy-sensing AMP-activated protein kinase (AMPK) (Kim et al., 2011). AMPK induces autophagy by alleviating the negative regulation by mTOR through the phosphorylation and activation of TSC2 (Inoki et al., 2003), an upstream negative regulator of mTOR, and via direct phosphorylation of Raptor (Gwinn et al., 2008), a subunit of the mTOR complex. Additionally, AMPK induces the formation of the ULK1 complex through direct phosphorylation of ULK1 at Ser 317 and Ser 777, which result in the activation of ULK1 kinase activity (Kim et al., 2011).

Following activation, ULK1 phosphorylates several downstream targets to initiate the autophagic process. Formation of the ULK1 multi-protein complex requires ULK1-mediated phosphorylation of ATG13 and the scaffold protein FIP200, thus resulting in the assembly of the ULK1-ATG13-FIP200-ATG101 complex (Jung et al., 2009; Zachari and Ganley, 2017). The ULK1 complex translocates to sites of autophagosome initiation, where it is responsible for the activation of a second essential autophagy effector protein complex, the phosphatidylinositol 3-kinase (PI3K) complex.

The PI3K complex is made up of the proteins VPS34, VPS15, Beclin 1, ATG14L, and AMBRA1. VPS34, a class III PI3K, is responsible for producing the phosphatidylinositol 3-phosphate (PI(3)P) that is required for autophagosome formation (Simonsen and Tooze, 2009). Enrichment in PI(3)P at specialized sites form the initiating autophagosome structure, known as the omegasome (Axe et al., 2008). The omegasome serves as a membrane platform, that remains in contact with the endoplasmic reticulum and with vesicles containing the transmembrane autophagy protein ATG9, to recruit the necessary subsequent core autophagy machinery that drive elongation of the autophagosome membrane (Orsi et al., 2012; Karanasios et al., 2016). The haploinsufficient tumor suppressor and critical autophagy effector protein Beclin 1 (the mammalian ortholog of yeast Atg6) is also a key member of the PI3K complex, and can regulate autophagy through its ability to interact with members of the anti-apoptotic BCL-2 family (Yue et al., 2003; Pattingre et al., 2005). BCL-2 directly interacts with Beclin 1 to negatively regulate autophagy, a process that is mediated by the BCL-2 adapter protein CISD2/NAF-1 at the endoplasmic reticulum (Chang et al., 2010).

Elongation of the autophagosome membrane is dependent on two ubiquitin-like conjugation steps (reviewed in Geng and Klionsky, 2008). The first involves the conjugation of the ubiquitin-like protein ATG12 to ATG5, which is catalyzed by ATG7 and ATG10. The ATG12-ATG5 conjugate forms a multi-protein complex with ATG16L1 and functions as an E3-like ligase. The second conjugation step is mediated by ATG7 and ATG3, which together with the ATG5-ATG12:ATG16L1 complex are responsible for conjugating phosphatidyl-ethanolamine to microtubule-associated protein 1 light chain 3 beta (MAP1LC3B, also known as LC3B), which has been proteolytically cleaved by ATG4 (LC3-I). Lipidated LC3B (referred to as LC3-II) is incorporated into the autophagosome membrane during elongation and is commonly used as an experimental marker to detect and quantify autophagosomes within cells (Kabeya et al., 2000).

Mature autophagosomes carrying cytoplasmic cargo are trafficked to lysosomes, and subsequent autophagosome-lysosome fusion is mediated by the Rab family of small GTPases, SNARE (soluble *N*-ethylmaleimide-sensitive factor attachment protein receptor) proteins, and membrane tethering proteins (reviewed by Nakamura and Yoshimori, 2017). The contents of the autophagolysosome are degraded by lysosomal acidic hydrolases and are exported back to the cytoplasm to be reused for metabolic processes.

## The Physiological Importance of Autophagy

**FIGURE 2:**
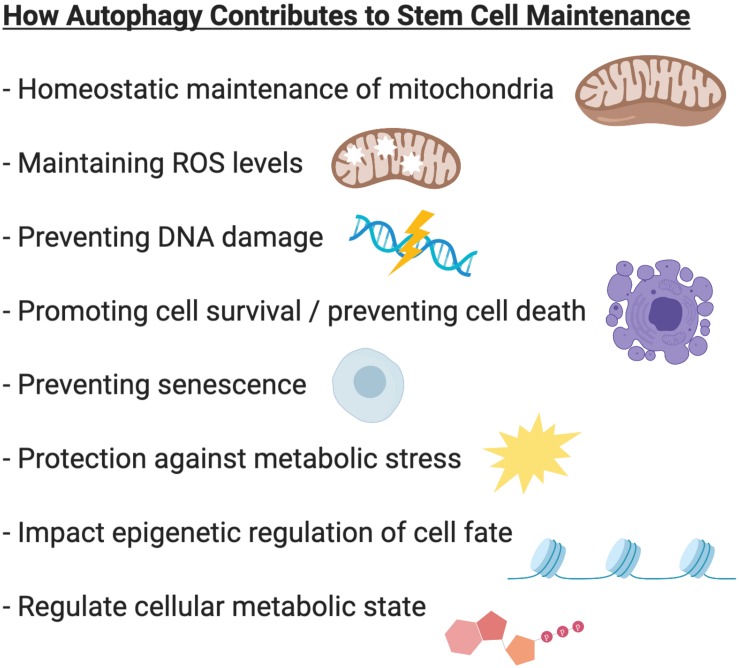
Summary of the role of autophagy in stem cell biology. In various stem cell systems, autophagy plays a critical role in stem cell maintenance. Autophagy regulates mitochondrial content to adapt to the metabolic requirements of the cell. Autophagy prevents accumulation of damaged mitochondria and the production of reactive oxygen species (ROS). Autophagy protects cells against metabolic stress and prevents genome instability, cell death and senescence. Through its ability to regulate epigenetic and metabolic programs, autophagy can also influence cell fate and regulate stem cell quiescence, activation, differentiation, and self-renewal.

**FIGURE 3:**
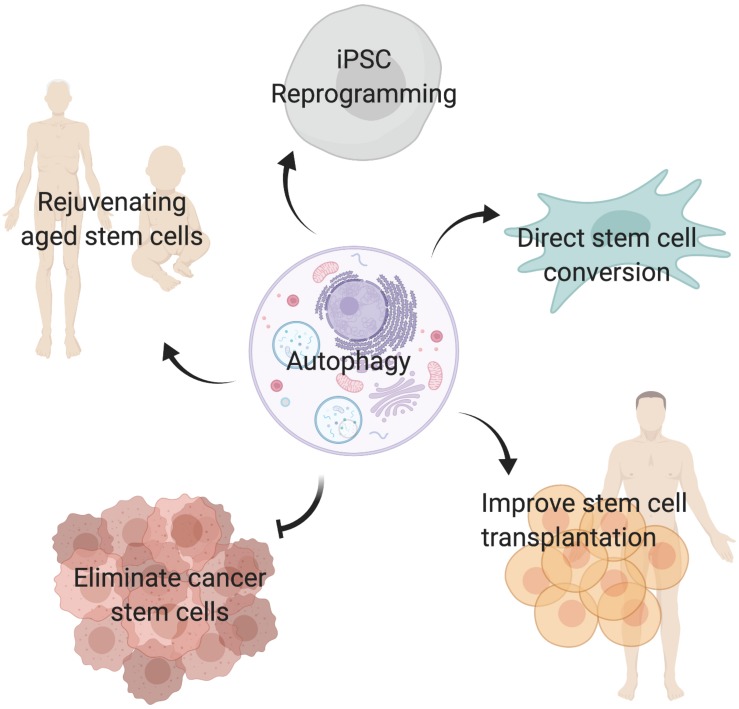
Targeting autophagy in stem cells. Inducing autophagy may improve aged stem cell function, promote reprogramming of somatic cells into induced pluripotent stem cells (iPSC), facilitate direct conversion of somatic cells into tissue specific stem cells, and improve stem cell transplantation therapies. In contrast, inhibiting autophagy in cancer stem cells may be a valid strategy to eliminate tumor initiating cells and prevent cancer treatment resistance.

Autophagy plays critical roles during embryonic development and is essential for maintaining cell survival, tissue homeostasis, and immunity. Importantly, dysfunctional autophagy has been linked to cancer, infectious diseases, neurodegeneration, muscle and heart diseases, as well as aging (Levine and Kroemer, 2008). Accumulating evidence demonstrates that autophagy is also critical for stem cell function. Adult stem cells are long-lived cells that are capable of undergoing differentiation to maintain tissue homeostasis and contribute to tissue repair, while also possessing the capacity for self-renewal to support the life-long maintenance of the stem cell reservoir. This review will focus on the emerging role of autophagy specifically in somatic stem cells (Figure 2) and discuss the merits of targeting autophagy as a therapeutic strategy for modulating stem cell function in regenerative medicine (Figure 3).

## Hematopoietic Stem Cells

Adult hematopoietic stem cells are multipotent stem cells residing in the bone marrow that give rise to both myeloid and lymphoid lineages to generate blood cells, a process termed hematopoiesis. Hematopoiesis supports the life-long daily generation of blood cells within the circulation that are in constant turnover. Thus, hematopoiesis is a highly orchestrated process that is required to maintain a balance between hematopoietic stem cell quiescence, activation, and differentiation. Importantly, perturbations in the regulation of hematopoiesis underlie blood disorders such as anemia (characterized by the loss of red blood cells) and leukemia (blood cancer caused by elevated numbers and defective maturation of leukocytes).

There are multiple lines of evidence supporting an essential role for autophagy in the maintenance and function of hematopoietic stem cells. Deletion of *Atg7* in the hematopoietic system resulted in a significant reduction in hematopoietic stem cells and progenitors of multiple lineages, indicating a critical role for autophagy in the maintenance of the hematopoietic stem cell compartment. Additionally, *Atg7*-deficient hematopoietic stem cells exhibited an accumulation of damaged mitochondria, increased levels of reactive oxygen species (ROS), and DNA damage (Mortensen et al., 2011).

Quiescent hematopoietic stem cells exhibit low oxidative phosphorylation levels and switch to a high oxidative phosphorylation metabolic state following their activation (Yu et al., 2013). Upon deletion of *Atg12* in hematopoietic stem cells, Ho et al. (2017) observed increased mitochondrial content accompanied by an activated metabolic state and enhanced myeloid differentiation, features that resemble an aging phenotype. Moreover, *Atg12*-deficient hematopoietic stem cells exhibited impaired self-renewal and regenerative potential, as determined by transplantation experiments. These findings indicate that autophagy plays an essential role in removing activated mitochondria as a mechanism for controlling oxidative metabolism to maintain hematopoietic stem cell quiescence and self-renewal. Interestingly, autophagy-dependent metabolic activation was linked to epigenetic reprogramming, as detected by changes in the DNA methylation profile of *Atg12*-deficient hematopoietic stem cells. Thus, basal autophagy is an important mediator of hematopoietic stem cell fate determination by controlling epigenetic-mediated mitochondrial content and metabolism (Ho et al., 2017).

While basal autophagy contributes to stem cell maintenance, hematopoietic stem cells are also capable of inducing the autophagy program in response to metabolic stress as a protective mechanism to prevent cell death. Hematopoietic stem cells express high levels of FOXOA3 (Warr et al., 2013), a transcription factor that targets and activates the transcription of pro-autophagy genes upon induction of the autophagy program (Mammucari et al., 2007). By maintaining high levels of FOXOA3, hematopoietic stem cells are poised for the rapid induction of autophagy genes in response to metabolic stress (Warr et al., 2013). In agreement with these results, *Foxo3a*-null aged mice exhibit a reduced hematopoietic stem cell pool, and loss of FOXOA3 results in impaired self-renewal capacity and defective maintenance of stem cell quiescence (Miyamoto et al., 2007).

In the context of aging, basal autophagy was found to be increased in a subset (around 30%) of aged hematopoietic stem cells compared to their younger counterparts (Ho et al., 2017). Augmented autophagy in a population of aged hematopoietic stem cells is attributed to the need for maintaining life-long survival of old hematopoietic stem cells within the aging bone marrow stem cell niche and conditions of reduced nutrient availability (Warr et al., 2013). It will be interesting to investigate why aged hematopoietic stem cells exhibit heterogeneity with respect to autophagic activity and to understand why hematopoietic stem cells have evolved an augmented autophagy response to aging in contrast to the dampened autophagy observed in other tissues (Rubinsztein et al., 2011).

Additionally, defective autophagy in the hematopoietic stem cell compartment can contribute to blood diseases. Defects in erythropoiesis, the process of generating red blood cells (erythrocytes), result in anemia, a blood condition that is characterized by the reduction of red blood cells. In erythroblasts, the autophagy pathway is critical for the selective clearance of mitochondria during erythroid differentiation. In a hematopoietic specific and inducible *Atg7* knockout mouse model (*Vav-iCre:Atg7^*fl/fl*^*), loss of *Atg7* resulted in severe anemia and eventual lethality at 8–14 weeks of age (Mortensen et al., 2010). Moreover, in an inflammatory cytokine-induced model of anemia in human hematopoietic stem/progenitor cells, it was found that TNFα-induction of anemia occurs via inhibition of autophagy in an mTOR-dependent manner (Orsini et al., 2019). Of note, not all hematopoietic lineages were equally affected by the loss of autophagy, suggesting distinct mechanisms in which autophagy contributes toward hematopoietic differentiation (Mortensen et al., 2010; Rožman et al., 2015).

## Neural Stem Cells

Somatic neural stem cells are multipotent self-renewing stem cells that reside in distinct niches within the subventricular zone of the lateral ventricles and subgranular zone of the hippocampal dentate gyrus of the adult brain. The progeny of neural stem cells, termed neural progenitor cells, can proliferate and differentiate into the three main cell types of the nervous system; neurons, astrocytes, and oligodendrocytes. While the importance of autophagy during embryonic development of the nervous system has been well-documented (reviewed in Boya et al., 2018; Casares-Crespo et al., 2018), the contribution of autophagy in adult neural stem cells and postnatal neurogenesis remain less well-defined. Of note, there is a lack of animal studies that employ genetic deletion of autophagy genes specifically in postnatal neural stem cells. Studies examining the impact of autophagy on the adult neural stem cell population have utilized animal models where the deletion of autophagy genes was performed during development. This makes it difficult to discern the effects of autophagy loss during postnatal neurogenesis that is independent from effects of autophagy loss in the embryo.

Similar to hematopoietic stem cells, transcriptional regulation of the autophagy program in neural stem cells is mediated by the transcription factor FOXO3. In *Foxo3*-deficient neural stem cells, defective autophagy results in the accumulation of protein aggregates (Audesse et al., 2019). Interestingly, another study found that lysosome-associated genes are highly upregulated in quiescent neural stem cells in contrast to activated neural stem cells that express high levels of proteasome-associated genes (Leeman et al., 2018). During quiescence, neural stem cells were found to contain an abundance of large lysosomes that contained insoluble protein aggregates. Inhibiting lysosome function resulted in the accumulation of these aggregates and impaired stem cell activation in response to growth factors. In contrast, activation of the autophagy pathway resulted in the clearance of protein aggregates and enhanced stem cell activation. Aged quiescent neural stem cells exhibited reduced lysosome content, increased amounts of protein aggregates and impaired stem cell activation that was reversible upon induction of lysosome activity. This study highlights the importance of lysosome-mediated protein degradation mechanisms (such as autophagy) in neural stem cells and also suggests a physiological role for protein aggregates in healthy neural stem cells (Leeman et al., 2018). It will be interesting to see if other stem cell systems exhibit similar protein aggregate content and lysosome dynamics in the transition between quiescence and activated states.

Also similar to the hematopoietic system, there is evidence supporting a role for mitochondria in the regulation of neural stem cell fate. While in hematopoietic stem cells, autophagy is critical for clearing metabolically active mitochondria to maintain quiescence (Ho et al., 2017), the role of mitochondria in neural stem cells is focused on the regulation of ROS-mediated signaling (Khacho et al., 2016). In neural stem cells, elongated mitochondria regulate self-renewal by maintaining low levels of ROS. During stem cell activation, the mitochondria transition to a fragmented state resulting in increased levels of ROS, which subsequently induce a gene expression signature that inhibits self-renewal while promoting commitment and differentiation (Khacho et al., 2016). Further studies are required to determine if autophagy, or even mitophagy (the selective autophagic degradation of mitochondria), plays a role in modulating the switch in mitochondrial dynamics between self-renewing and differentiating neural stem cells.

In another study, FIP200, a component of the ULK1-autophagy activating complex was deleted in neural stem cells in mice using *GFAP-Cre*, where expression of Cre recombinase is under the control of human glial fibrillary acidic protein (GFAP). Developmental deletion of *FIP200* resulted in increased mitochondrial content and ROS levels in postnatal neural stem cells, which lead to progressive depletion of the adult neural stem cell pool (Wang C. et al., 2013). Intriguingly, the same group deleted the autophagy genes *Atg5* and *Atg16L1* using the same *GFAP-Cre* deletion strategy and found no impact on neural stem cell maintenance (Wang et al., 2016).

With respect to differentiation, *in vitro* neurosphere assays with *FIP200*-*null* neural progenitor cells indicated defects in self-renewal and neural differentiation (Wang C. et al., 2013). Moreover, GFAP-mediated deletion of *FIP200* resulted in increased infiltration of microglia immune cells into the subventricular zone, which inhibited differentiation of neural stem cells. Thus, in addition to a cell autonomous role for FIP200 in neural stem cells, FIP200 also influences neural differentiation via extrinsic mechanisms to restrict microglia infiltration (Wang et al., 2017). Additional *in vitro* studies in primary rat hippocampal neural stem cells have indicated that autophagic flux increases during neural differentiation. Depletion of the autophagy genes *Atg7*, *LC3*, and *p62* using lentiviral shRNA and CRISPR/Cas9 approaches had an inhibitory effect on astrogenesis (Ha et al., 2019). These results collectively demonstrate that autophagy plays a contributing role in neural differentiation.

In addition, autophagy has also been shown to promote survival and prevent cell death in neural stem cells. Adult neural stem cells isolated from *Ambra1* and *Beclin1* heterozygous mice exhibited reduced cell survival and impaired neural differentiation *in vitro* (Yazdankhah et al., 2014). Additional studies using a retroviral strategy to delete *Atg5* in dividing neural progenitor cells in the adult brain found that *Atg5* is critical for the survival of proliferating neural progenitor cells. Loss of *Atg5* in neural progenitor cells resulted in cell death that was dependent on the expression of BAX, a pro-apoptotic member of the BCL-2 family. Interestingly, the subpopulation of *Atg5-null* progenitor cells that survive are able to undergo differentiation and generate functional neurons despite a delay in their maturation (Xi et al., 2016).

Of note, although autophagy is typically viewed as a survival mechanism, induction of autophagy can also result in cell death. Primary rat hippocampal neural stem cells undergo autophagic cell death in response to metabolic stress induced by insulin withdrawal and oxygen-glucose deprivation *in vitro* (Ha et al., 2017; Chung et al., 2018). Additionally, autophagy-mediated cell death programs also occur *in vivo* in mice subjected to chronic restraint stress. Neural stem cells are protected against autophagy-mediated cell death in mice where *Atg7* is conditionally deleted (using a *Nestin-CreERT2:Atg7^*fl/fl*^* mouse model) (Jung et al., 2020). Thus, the contribution of autophagy in neural stem cells can be beneficial and detrimental depending on the context in which autophagy is being induced.

Dysfunctional autophagy has been shown to contribute to the development of neurodegenerative diseases, including Alzheimer, Parkinson and Huntington disease (reviewed in Menzies et al., 2015). In the absence of autophagy, persistence and defective clearance of misfolded proteins and protein aggregates result in progressive neurodegeneration. The contribution of autophagy in neural stem cells within the context of neurodegeneration is not well-understood. Loss of either *Atg5* or *Atg7* in neural cells under the control of the Nestin promoter (*Nestin-Cre*) were shown to cause neurodegenerative disease in mice (Hara et al., 2006; Komatsu et al., 2006). As these models perturb the autophagy pathway during embryonic neural development, it will be critical to determine how disrupting autophagy in postnatal neural stem cells contributes to neurodegeneration during adulthood, which would be more relevant to aging-related neurodegeneration. Understanding the role of autophagy in adult neural stem cells is essential for the development of strategies that aim to simulate endogenous repair mechanisms within the neural stem cell and progenitor population for the treatment of neurodegenerative diseases.

## Muscle Stem Cells

In skeletal muscle tissue, muscle homeostasis and regeneration are mediated by resident muscle stem cells, which are also known as satellite cells. Short-term caloric restriction in mice was found to increase satellite cell number and improve muscle regeneration and satellite cell transplantation efficiency (Cerletti et al., 2012), suggesting that nutrient deprivation-induced autophagy may enhance muscle stem cell function.

In stark contrast to stem cells in the hematopoietic system, aged muscle cells were reported to have impaired basal autophagy levels. Using GFP-LC3 mice, quiescent satellite cells from young mice were found to exhibit robust levels of autophagic flux which was reduced in an age-dependent manner in satellite cells from old and geriatric mice (García-Prat et al., 2016). Moreover, stimulating autophagy in old satellite cells improved stem cell function, as determined by transplantation and engraftment assays, and prevented aging-induced entry into senescence. Genetic deletion of *Atg7* in satellite cells resulted in a loss of the satellite cell pool, indicating that autophagy is important for quiescent satellite cell maintenance. Residual satellite cells were found to express markers of senescence and DNA damage, which resembled an aging phenotype, and were unable to participate in muscle regeneration. *Atg7*-deficient satellite cells, similar to old satellite cells, exhibited defective mitophagy and increased levels of ROS. Altogether, these results indicate that autophagy in muscle stem cells is required to prevent stem cell senescence and that inducing the autophagic pathway may improve muscle stem cell function in the context of aging (García-Prat et al., 2016).

Of note, autophagy genes in muscle stem cells were found to be expressed in an oscillatory fashion, expressing higher levels of autophagy and lysosome markers during the day compared to at night. The rhythmic expression of autophagy genes is abrogated in aged muscle stem cells, which can be rescued by subjecting mice to a caloric restriction diet (Solanas et al., 2017). Intriguingly, supplementing nicotinamide adenine dinucleotide (NAD^+^) in the diet of aged mice lead to an improvement in aged muscle stem cell function and increased life span. NAD^+^ repletion enhanced mitochondrial function and prevented muscle stem cell senescence (Zhang H. et al., 2016). It will be interesting to examine if NAD^+^-mediated improvement in muscle stem cell function occurs via stimulation of the autophagy pathway.

On a different note, another study reported that autophagy is induced during satellite cell activation (Tang and Rando, 2014) to help satellite cells adapt to the increased metabolic demands associated with the switch from quiescence to activation (reviewed by Nguyen J.H. et al., 2019). In contrast to the study by García-Prat and colleagues, Tang and colleagues also used GFP-LC3 mice and reported undetectable levels of autophagic flux in quiescence satellite cells and increased autophagic flux in activated satellite cells. Notably, Tang and colleagues also found that inhibition of autophagy lead to a delay in satellite cell activation and did not result in permanent cell cycle arrest. The delay in cell cycle entry was attributed to insufficient ATP levels to support the cellular energy demands associated with stem cell activation (Tang and Rando, 2014). In another study, Fiacco and colleagues performed immunostaining for the autophagy marker LC3 on muscle cross-sections and were unable to detect LC3 expression in resting muscle. Following muscle damage, however, they observed an induction in LC3 expression in regenerating muscle. LC3 immunostaining in combination with myogenic markers for commitment and self-renewal revealed that committed myogenic progenitors (expressing the myogenic commitment factor MYOD) were mostly positive for LC3 expression and not the self-renewing population (PAX7-expressing cells), suggesting that autophagy plays a contributing role during myogenic differentiation (Fiacco et al., 2016). Thus, further investigation is required to resolve these contrasting and potentially overlapping results concerning the role of autophagy in muscle stem cell biology.

Muscle stem cell dysfunction is one of the contributing factors in Duchenne muscular dystrophy (Dumont et al., 2015; Chang et al., 2018), a severely debilitating muscle degenerative disease that results from the genetic loss of the dystrophin gene *Dmd* (reviewed in Chang et al., 2016). Impaired autophagy has been reported in muscles from both dystrophic mouse models and Duchenne muscular dystrophy patients (Spitali et al., 2013; Fiacco et al., 2016). Although there is some indication that the expression of LC3 is reduced in committed myogenic progenitors in muscle biopsies from Duchenne muscular dystrophy patients (Fiacco et al., 2016), it remains unclear how autophagy is impaired specifically in the dystrophic muscle stem cell population and how this may contribute to disease progression.

## Induced Pluripotent Stem Cells

Cellular reprogramming of differentiated somatic cells into undifferentiated pluripotent stem cells has been made possible through the pioneering work of Shinya Yamanaka. Ectopic expression of the pluripotency transcription factors Oct4, Sox2, Klf4, and c-Myc permits the resetting of somatic cells into undifferentiated cells that resemble embryonic stem cells, termed induced pluripotent stem cells (iPSCs) (Takahashi and Yamanaka, 2006). Interestingly, autophagy has been shown to be required for the quality and efficiency of the somatic reprogramming process.

In particular, autophagy is upregulated during the early stages of reprogramming, which corresponds to an inhibition in mTOR signaling (Wang S. et al., 2013). Inhibitors of mTOR, such as rapamycin, were found to increase the efficiency of the reprogramming process, while activating mTOR impeded reprogramming (Chen et al., 2011; He et al., 2012). Of note, mTOR inhibition at later stages was found to inhibit reprogramming, indicating that early upregulation of autophagy is transient and its downregulation is required to complete the reprogramming process (Wang S. et al., 2013).

It has been proposed that autophagy may promote reprogramming by preventing cell death and cellular senescence, which impede the reprogramming process. However, an alternative explanation for the role of autophagy during reprogramming may be the ability to mediate mitochondrial remodeling. Indeed, stem cells in general have less mitochondria than their differentiated counterparts, in accordance with a heavy reliance on glycolysis rather than oxidative phosphorylation for energy production in stem cells (Ito and Suda, 2014). Metabolic transitions from oxidative phosphorylation to glycolysis during reprogramming have also been observed (Folmes et al., 2011). Several autophagy and mitophagy genes play a critical role in mitochondrial homeostasis and clearance of somatic mitochondria during reprogramming including ATG3, PTEN-induced putative kinase 1 (PINK1), and BCL2-interacting protein 3 like (BNIP3L) (Liu et al., 2016; Vazquez-Martin et al., 2016; Xiang et al., 2017). However, the molecular regulation of autophagy and/or mitophagy-dependent remodeling during somatic reprogramming require further elucidation.

## Cancer Stem Cells

Cancer stem cells, also known as tumor-initiating cells, are a subpopulation of cells within a tumor that are highly tumorigenic and able to hyperactively self-renew and differentiate to generate the different cell types within a heterogenous tumor (Reya et al., 2001). Cancer stem cells were first identified in acute myeloid leukemia (AML) as a population of cells that were able to regenerate AML when transplanted into immune-compromised mice (Bonnet and Dick, 1997). Since the initial discovery of a tumor-initiating cell with self-renewing and differentiating capabilities, cancer stem cells have been identified in many other types of cancers including cancers of the brain, breast, lung, pancreas, colon, ovary, and skin. A key property of cancer stem cells is their ability to maintain a quiescent dormant state and resist anti-cancer treatment, which is a significant contributing factor to tumor recurrence following therapy.

The role of autophagy in cancer is complex as there is evidence demonstrating that autophagy can both promote and inhibit tumorigenesis (White, 2012). On one hand, autophagy has been established as an essential cytoprotective mechanism that safeguards cells against oncogenic stresses such as oxidative stress and DNA damage, thus preventing chromosomal instability and tumorigenesis. Autophagy also protects cells from undergoing necrotic cell death and triggering inflammatory responses, which also contribute to oncogenesis. On the other hand, cancer cells, which are subject to conditions of hypoxia and reduced nutrient availability, upregulate basal autophagy levels in order to promote their own survival. Additionally, induction of autophagy in cancer cells can also prevent the effectiveness of anti-cancer treatment. Altogether, these findings indicate that the contribution of autophagy in cancer is highly complex and context-dependent.

Given the evidence demonstrating that autophagy is essential for stem cell maintenance in multiple stem cell types (Figure 2), it is not surprising that autophagy also plays critical roles in cancer stem cell maintenance and function. In breast cancer, cancer stem cells are described as a population of CD44^high^/CD24^–/low^ expressing cells that express high levels of aldehyde dehydrogenase 1 (ALDH1). Functionally, breast cancer stem cells possess the ability to regenerate breast cancer tumors *in vivo* upon transplantation into the mammary fat pad of immunodeficient mice and can form floating spheres, termed mammospheres, when cultured in serum-free media *in vitro* (Al-Hajj et al., 2003; Dontu et al., 2003; Ginestier et al., 2007). When comparing breast cancer stem cell-enriched mammospheres to heterogenous cultures of adherent breast cancer cells, one study found that autophagic flux and Beclin 1 expression are higher in mammospheres. As well, both proliferation and maintenance of mammospheres were dependent on Beclin 1 expression (Gong et al., 2013). Mechanistically, a subsequent study found that autophagy supports the survival of breast cancer stem cells by mediating the secretion of interleukin-6 (IL6), a cytokine that is important for breast cancer stem cell maintenance (Maycotte et al., 2015). In transplantation assays, depletion of Beclin 1 in breast cancer stem cells inhibited xenograft tumor formation in immunodeficient mice. Interestingly, Beclin1 deletion in adherent breast cancer cells had the opposite effect. Tumor generation was enhanced upon transplantation of Beclin 1-deleted adherent breast cancer cells (Gong et al., 2013), which supports the role of Beclin 1 as a general tumor suppressor (Yue et al., 2003). These distinct differences in the requirement for Beclin1 in tumor formation in differentiated cancer cells versus cancer stem cells may provide an explanation as to why autophagy both promotes and inhibits tumorigenesis in cancer.

In chronic myeloid leukemia (CML), CD34^+^ CML stem and progenitor cells expressed increased levels of autophagy genes. Specifically, ATG4B was highly upregulated at both the transcript and protein levels, indicating that ATG4B may be a potential biomarker and target for CML cancer stem cells. Interestingly, leukemic stem cells that were refractory to treatment with imatinib mesylate, a potent tyrosine kinase inhibitor, expressed higher levels of ATG4B in comparison to cells that responded well to imatinib. Knockdown of ATG4B impaired cell proliferation, colony-formation capacity and cell survival of CML stem cells. Importantly, depletion of ATG4B also sensitized CML cancer stem cells to imatinib treatment, which indicates that inhibiting autophagy in combination with imatinib would have therapeutic potential in the treatment of CML (Rothe et al., 2014).

Similarly, in AML, genetic deletion of *Atg5* and *Atg7*, delayed tumor progression of leukemic mice and reduced the function and survival of AML-initiating stem cells. In the absence of autophagy, AML stem cells exhibited increased mitochondria, enhanced levels of ROS, and a higher frequency of apoptotic cell death. Moreover, in the absence of *Atg7*, *in vivo* treatment of leukemic mice with the chemotherapeutic drug cytarabine, commonly known as AraC, resulted in selective targeting of AML-initiating cells and greatly enhanced the potency of AraC treatment (Sumitomo et al., 2016). These findings demonstrate that autophagy plays a critical role in cancer stem cell survival, which contribute to cancer therapeutic resistance.

As well, a recent study found that human AML stem cells exhibited high expression of the mitochondrial fission 1 (FIS1) protein and unique mitochondrial morphology. AML stem cells were highly dependent on FIS1-mediated mitophagy that is induced by constitutively active AMPK. Importantly, disruption of either FIS1 or AMPK in AML stem cells induced myeloid differentiation and cell cycle arrest, and greatly reduced engraftment potential (Pei et al., 2018). Additionally, high expression of the autophagy receptor p62 was associated with poor prognosis in human AML. Specifically in leukemia cells, p62 interacts with damaged mitochondria to mediate their clearance via mitophagy. Loss of p62 impaired leukemia cell proliferation and survival, and delayed leukemia development in an AML mouse model (Nguyen T.D. et al., 2019). These findings indicate a specific role for mitophagy in the self-renewal and maintenance of AML stem cells and provide the rationale for developing compounds that target selective autophagy in leukemia.

## Modulating Autophagy in Stem Cells

While it appears that targeting autophagy to inhibit the autophagy-mediated cell survival properties in cancer stem cells may hold promise for anti-cancer therapy, the importance of autophagy in maintaining normal stem cell function suggest that inducing autophagy may have therapeutic potential for regenerative medicine (Figure 3). Certainly within the context of aging, stimulation of autophagy via genetic and pharmacological approaches in aged stem cells have improved their regenerative capacity and function (García-Prat et al., 2016; Leeman et al., 2018).

Intriguingly, few studies have examined the direct impact of autophagy modulation in stem cell transplantation therapy. Stem cell transplantation is an exciting approach in regenerative medicine as it allows delivery of functional stem cells to repair the damaged or degenerative tissue with the potential of repopulating the stem cell niche and providing a long-term means of correcting the degenerative phenotype. However, various limitations have impeded the use of stem cell transplantation within the clinic including difficulty in expanding stem cell cultures *ex vivo*, poor survival of engrafted stem cells, preferred differentiation and limited self-renewing capacity of transplanted cells, as well as the risk of causing graft-versus-host disease. As an example, hematopoietic stem cells isolated from human umbilical cord blood hold tremendous potential for hematopoietic stem cell transplantation therapy. However, widespread clinical use of cord blood as a primary source of stem cells is limited by the low yield of hematopoietic stem and progenitor cells within each cord blood unit. In a recent study, *ex vivo* culturing of human cord blood-derived hematopoietic stem cells in the presence of *N*-(4-hydroxy-phenyl) retinamide (4HPR), an inhibitor of the sphingolipid enzyme DEGS1, enhanced self-renewal of a specialized population of long-term hematopoietic stem cells (Xie et al., 2019). 4HPR treatment increased long-term repopulating cell frequencies following serial xenotransplantation into immune deficient mice compared to untreated cells. Intriguingly, 4HPR-mediated improvement of hematopoietic stem cell function was attributed to activation of quality control proteostasis programs including autophagy and the unfolded protein response. Thus, the identification of compounds that can increase stem cell expansion and simultaneously preserve stem cell self-renewing functions show great promise for the future of stem cell transplantation approaches (Fares et al., 2014; Xie et al., 2019).

Moreover, directed differentiation of iPSCs or direct reprogramming from somatic cells into the various types of tissue-specific stem cells provides the possibility of generating an unlimited supply of stem cells. In addition, correction of genetic defects via CRISPR/Cas9-mediated approaches in patient cells along with reprogramming avoids the problem of transplantation-related immune complications. Interestingly, SMER28, a small molecule enhancer of rapamycin, which enhances autophagy and has been shown to induce clearance of autophagy substrates in fly models of Huntington’s disease (Sarkar et al., 2007), was identified in a small molecule screen to improve the efficiency of neural stem cell reprogramming (Zhang M. et al., 2016). It will be interesting to determine the molecular mechanism in which autophagy contributes to reprogramming and examine the benefits of modulating autophagy in reprogramming protocols.

## Concluding Remarks

Historically, autophagy has been viewed as a non-selective housekeeping process that is upregulated during conditions of nutrient deprivation to provide the cell with an alternative source of energy. Our understanding of autophagy has evolved and increasing evidence demonstrates that autophagy is an active program that controls the metabolic status of the cell. In stem cells, autophagy is emerging as an important mechanism for the homeostatic maintenance, function and survival of long-lived stem cells. Autophagy can also influence cell fate decisions through its ability to influence mitochondrial content, energy production, and epigenetic programming (summarized in Table 1). Particularly in the context of aging and degenerative conditions that involve the decline of stem cell regenerative capacity, autophagy plays key roles in protecting stem cells against cellular stress and is transpiring as a viable target in regenerative medicine.

**TABLE 1:** The role of autophagy in different stem cell types.

Stem cell	Autophagy contribution
Hematopoietic stem cell	Quiescence and maintenance
	Self-renewal
	Metabolism
	Protection against metabolic stress
Neural stem cell	Quiescence and maintenance
	Self-renewal
	Clearance of protein aggregates
	Survival
	Differentiation
Muscle stem cell	Quiescence and maintenance
	Prevent senescence
	Activation
	Metabolism
Induced pluripotent	Reprogramming
stem cell	Cellular remodeling
Cancer stem cell	Self-renewal
	Survival

## Author Contributions

NC conceptualized and wrote the review.

## Conflict of Interest

The authors declare that the research was conducted in the absence of any commercial or financial relationships that could be construed as a potential conflict of interest.
